# Metabolomics Provides A Novel Interpretation of the Changes in Main Compounds during Black Tea Processing through Different Drying Methods

**DOI:** 10.3390/molecules26216739

**Published:** 2021-11-08

**Authors:** Fei Ye, Xiaoyan Qiao, Anhui Gui, Shengpeng Wang, Panpan Liu, Xueping Wang, Jin Teng, Lin Zheng, Lin Feng, Hanshan Han, Shiwei Gao, Pengcheng Zheng

**Affiliations:** 1 Institute of Fruit and Tea, Hubei Academy of Agricultural Sciences, No.10 South Lake Avenue, Hongshan District, Wuhan 430064, China; yf421@163.com (F.Y.); guianhui@tricaas.com (A.G.); wwsspp0426@163.com (S.W.); liuppitea@163.com (P.L.); wangxueping79-79@163.com (X.W.); jobbase@163.com (J.T.); caozi20121117@163.com (L.Z.); zuotianyujintian@163.com (L.F.); 2 Guangdong Provincial Key Laboratory of Tea Plant Resources Innovation and Utilization, Tea Research Institute, Guangdong Academy of Agricultural Sciences, No.6 Dafeng Avenue, Tianhe District, Guangzhou 510665, China; qiaoxiaoyan@tea.gdaas.cn; 3 Mu Lan Tian Xiang Co., Ltd., Huangpi District, Wuhan 432200, China; It386574909@163.com

**Keywords:** black tea, metabolite, E’cha NO1, drying methods, non-targeted metabolomics

## Abstract

This study aimed to compare the effect of hot roller (HR) drying and hot air (HA) drying on the sensory evaluation, chemical quality, antioxidant activity, and metabolic profile of Yihong Congou black tea processed from E’cha NO1. The Yihong Congou black tea dried with HA obtained higher sensory scores and better chemical qualities such as the hue of tea brew color (*a* and *b*), content of theaflavins, thearubigins, water extract, free amino acids, tea polyphenol, and the ratio of polyphenol to amino acids as well as higher antioxidant capacities compared to that dried with HR. The HA drying tea increased the contents of volatile compounds that had positive correlation with sweet and flowery flavor, while the HR drying tea increased the contents of volatile compounds related to fruity flavor. Moreover, non-targeted metabolomics data indicated that the levels of most free amino acids significantly increased, while the levels of most soluble sugars reduced in the HA drying method compared to the HR drying method. The metabolic analysis was also consistent with the above results and revealed that D-ribose and gallic acid were the main characteristic metabolites of HA drying. Our results could provide a technical reference and theoretical guide to processing a high quality of Yihong Congou black tea.

## 1. Introduction

Tea (*Camellia sinensis* L.) is one of the most traditional beverages consumed worldwide due to its health benefits, satisfactory taste, and aroma [1]. Unlike green tea (unfermented) and oolong tea (partially fermented), black tea (fermented), attributed to its appealing flavors and beneficial health effects [2,3], has become one of the most popular beverages in the world, accounting for up to 78% of tea consumption worldwide [4]. Black tea has a series of potent bioactivities such as antioxidant, anti-inflammatory, anti-mutagenic, anti-cancer, and increase in psychomotor performance activities [5,6,7]. In China, Congou black tea with thin and strip-sharped whole-leaves is preferable to broken black tea [8]. Yihong Congou black tea, which is one of the top three Chinese Congou black teas, has high quality and taste flavor.

As far as Congou tea processing is concerned, researchers have focused on the improvement in sensory and physico-chemical quality of Yihong Congou black tea [9]. There are four steps in Yihong Congou black tea manufacturing, namely withering, rolling, fermentation and drying, among which drying plays an important role in fragrance formation and quality fixation of famous high-quality tea. There has been much research involved in withering and fermentation, however, little is known about Yihong black tea drying research [10,11].

Drying is an important part of black tea processing, which reduces water content, promotes the formation of aroma, taste, and color, and extends shelf life [12]. Currently, there are several drying methods, for instance, hot air drying, hot roller drying, microwave drying, far-infrared drying, halogen lamp drying, pulsed electric drying, pan-fired drying, light-wave drying, vacuum drying, the combination of these drying method, and so on. The above drying methods have both advantages and disadvantages. Hot air drying, a traditional drying method, operates simply and has low costs, but is time consuming [13,14]. The pan-fired drying methods have been in large-scale applications in green tea, and oolong tea processing [15]. Some different new drying methods such as pulsed electric drying, far-infrared drying, microwave drying, light-wave drying, vacuum-microwave drying and light-wave-microwave combination drying, have been extensively used for drying vegetables, fruits, and natural products. However, in fact, as far as we know, these new drying methods have not been in popular application in the quality improvement of Congou black tea [16,17]. However, the sophisticated drying process, in which numerous reactions took place simultaneously, was often characterized by determining only a small amount of compounds in previous studies due to the limitations of the analytical method.

Based on the above analysis, a more comprehensive investigation is needed to explore the dynamic variations of the tea compounds’ metabolic profile. Therefore, this study aimed to conduct a sensory evaluation and analyze the dynamic changes in chemical qualities, aroma component volatiles, and antioxidant capacity of Yihong black tea processed by the HA and HR drying methods. These findings could provide production guidance to manufacture stable and high-quality tea, and enhance the production efficiency.

## 2. Materials and Methods

### 2.1. Experimental Materials

Fifty kilograms of two leaves and one bud of tea clone “E’cha NO1” were plucked from a tea garden (30°28′97″ N, 114°16′47″ E, *Camelia sinensis* cv E’cha NO1) in Jinshuizha Village (Wuhan, China) on 3 May 2019.

### 2.2. Methods of Yihong Congou Black Tea Processing Procedures

Fresh tea leaves were plucked, withering 16 h at a thickness of 8 cm and 28 °C with an air flow of 4.89 m/s until 100 g of leaves contained 60 g of water. During withering, the leaves were shuffled every 4 h, with the ambient air regulated by a blower (on for 1.5 h, then off for 1.5 h, and on again). The withered leaves were subjected to rolling for 90 min in a roller machine (6CR-55, Zhejiang Shang Yang Co., Ltd., Quzhou, China). Subsequently, the rolled leaves were fermented in an artificial climate box (RXZ-328A, Changzhou City Solid Germany Instrument Co., Ltd., Changzhou, China) at 28 °C and 90% relative humidity for 6 h with air flow. Finally, the fermented leaves were treated with five repeats.

*Hot air drying*: The fermented leaves (about 1 cm thickness) were spread on the drying tray of a hot air drying machine (6CH-10, Zhejiang Shang yang Co., Ltd., Quzhou, China) at 120 °C for 25 min, followed by cooling outside the drying machine for 1.5 h, and finally dried at 90 °C for about 100 min.

*Hot roller drying*: The fermented leaves (about 8 kg) were placed in the hot roller drying machine (6CST-90B, Fujian Jiayou Machinery Intelligent Technologies Inc., Quanzhou, Fujian, China) and heated at 135 °C for 30 min, followed by 1 h cooling outside the oven, and then heated again at 90 °C for 30 min.

### 2.3. Sensory Evaluation

The tea sensory quality was assessed by five professional tea tasters from the Institute of Fruit and Tea, Hubei Academy of Agricultural Sciences, China. The description and scores of the tea samples were assessed according to the national standards for the methodology of the sensory evaluation of tea (GB/T 23376-2018) and tea vocabulary for sensory evaluation (GB/T14487-2017). Briefly, tea samples (3 g) were extracted with 150 mL freshly boiled distilled water for 5 min. Tea infusions were individually presented in white porcelain bowls and tea samples were blind-coded with random numbers. Then, panelists were instructed to smell and drink the tea infusions and pause for 30 s between the different samples [18]. Each sample was assessed three times through blind evaluation.

### 2.4. Determination of Tea Color, Physico-Chemical Quality and Antioxidant Capacity

The color of the tea, brew, and infused tea was determined by the hue determination [17]. Moisture content was analyzed by using the 120 °C-drying method (GB/T 8304-2013). The content of amino acids was analyzed by using the ninhydrin colorimetric method (GB/T5009. 124-2003). The content of total tea polyphenols was analyzed by using the iron tartrate colorimetric method (ISO14502-1:2005). Soluble sugar content was analyzed using the anthrone colorimetric method [19]. The contents of theaflavins, thearubigins, and theabrownins were determined by systematic analysis [20]. The antioxidant capacity was determined by the eliminating rate of DPPH free radicals at various concentrations [21].

### 2.5. Analysis of Tea Volatiles

A solid-phase micro-extraction (SPME) fiber was exposed for 10 min in the injection port of the gas chromatography (GC) instrument at 280 °C to remove any remaining volatiles from the fiber before each extraction. A total of 3.0 g of the dry tea sample was added to a 100 mL vial sealed with silicone septa and infused with 30 mL of boiling water, then, 20 μL of the internal standard solution (ethyl caprate) was added immediately. The vial was kept in a water bath at 50 °C for 10 min to equilibrate and the SPME fiber was exposed for 50 min to the head space while the sample was maintained at 50 °C. The fiber was then placed in the GC injector port and thermally desorbed at 240 °C for 3 min.

GC-MS analysis was performed on an Agilent 7890A GC interfaced with an Agilent 5975C MSD Ion Trap MS. A DB-5MS capillary column (30 m × 0.25 mm × 0.32 µm) was used for separating. The GC oven temperature conditions were as follows: 50 °C (held for 5 min) initially, increased to 180 °C (held for 2 min) at a rate of 3 °C/min, and finally, increased to 250 °C (held for 3 min) at a rate of 10 °C/min. Helium (percentage purity >99.999%) was used as the carrier gas at a constant flow rate of 1.0 mL/min. The MS was operated in the EI mode at an electronic energy of 70 eV. The injector and ion source temperature were 240 °C and 230 °C, respectively, and the MS was scanned at a range of 35–400 AMU. Compounds were tentatively identified using the National Institute of Standards and Technology (NIST) library (14.L). Additionally, the linear retention indices (RI) were calculated using n-paraffins C7–C40 as external references as described previously [22,23]. The concentration of the volatiles was calculated in µg/L based on an internal standard solution.

### 2.6. Analysis of the Composition of Yihong Congou Black Tea through Non-Targeted Metabolomics

To gain an overview of the metabolic profile, and to also confirm the results of the sensory quality, physico-chemical quality, and volatile compounds of Yihong Congou black tea processed by the HA and HR drying methods, non-targeted metabolomics was carried out in the following steps. The experiments were performed on an ultrahigh performance gas chromatography system (Agilent 6890A/5973C GC-MS). About 50 mg of feces was applied to the extraction procedure and extracted with 800 μL of methanol, then, 10 μL of internal standard (2.8 mg/mL, DL-o-chlorophenylalanine) was added. Chromatographic separation of the tea metabolome was carried out on the column (Agilent J&W Scientific, DB-5 ms, 30 m × 0.25 mm × 0.25 μm). The injection conditions were as follows: injector temperature 280 °C, ion source temperature 230 °C, quadrupole rod temperature 150 °C, helium (high purity) >99.999%, injection mode splitless, injection volume 1.0 μL. The column temperature condition: was held at 70 °C (held for 2 min), increased to 200 °C at a rate of 10 °C/min, then increased to 280 °C (held for 6 min) at a rate of 5 °C/min. The column effluent was fully scanned in the mass ranged 50–550 m/z.

Feature extraction was then performed on the data and preprocessed with XCMS in R software, and then normalized and edited into a two-dimensional data matrix by Excel 2010 software including retention time (RT), mass-to-charge ratio (MZ), observations (samples), and peak intensity. A total of 966 features were collected in this experiment, then multivariate analysis (MVA) was performed on the data after editing using SIMCA-P 13.0 software (Umetrics AB, Umea, Sweden).

### 2.7. Statistical Analysis

The results were expressed as a mean of three measurements for the analytical determination. The analysis of significant differences between means was determined by one-way ANOVA (Duncan’s multiple range tests) using SPSS 23.0 (Demo version, Armonk, NY, USA). Figures were made by Origin 8.0 software (Demo version, Northampton, MA, USA). PCA, PLS-DA, and OPLS-DA were conducted using SIMCA-P 13.0 software (Umetrics, Umea, Sweden). HCA was generated by the Multi Experiment Viewer (MEV) 4.9.0 (Oracle Corporation, Redwood Shores, CA, USA).

## 3. Results

### 3.1. Effect of Drying Methods on Congou Black Tea Sensory Quality and Color Quality

The sensory evaluation results of Yihong Congou black tea processed by two different drying methods are listed in Table 1. The drying methods caused a distinct impact on appearance color, aroma, and taste of the Congou black tea, but little on infused leaf. The HA dried Congou black tea showed curly shaped, black bloom color, while the HR dried Congou black tea showed a curly tight and heavy, greyish black color. The color qualities results of Yihong Congou black tea processed by two different drying methods were listed in Table 2. The drying methods also caused a distinct impact on the hue of tea color. The above results indicated that HA drying could better retain the color of dried Congou black tea. Furthermore, in contrast with HA drying, the dry tea streak and infused leaf of black tea could be improved by the HR drying method. Thus, we speculated that the HR drying technology was responsible for the formation of the appearance and flavor characteristics in Yihong Congou black tea.

### 3.2. Effect of Drying Methods on Tea Physico-Chemical Quality and Antioxidant Capacity

**Figure 1:**
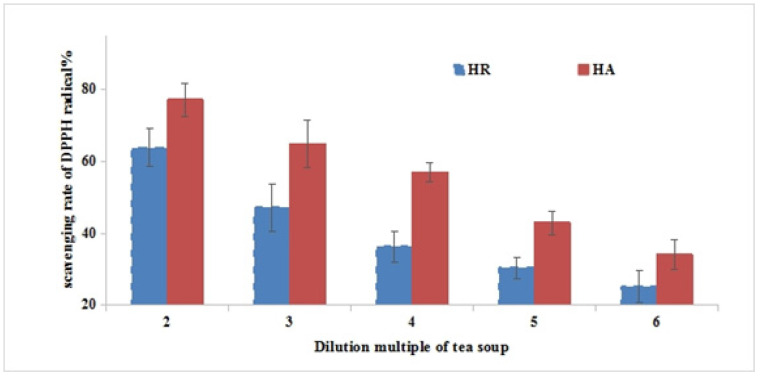
Scavenging rates of Congou black tea processed by hot air (**HA**) and hot roller (**HR**) treatments at various concentrations on DPPH free radicals.

The chemical compositions and antioxidant capacity of Yihong Congou black tea treated with HA drying and HR drying methods are presented in Table 3 and Figure 1. The HA treatment was significantly higher in water extracts, amino acids, polyphenol, theaflavins, thearubigins, theabrowns content, and antioxidant capacity than that of HR treatment, except for soluble sugar. The results suggested that the HA drying probably promoted the formation of theaflavins (TF_S_), thearubigins (TR_S_), and theabrownins (TB_S_), while the HR drying promoted the higher content of soluble sugar, which was beneficial to improving the taste.

### 3.3. Effect of Drying Methods on Yihong Congou Black Tea Volatile Compounds

Drying methods had a great effect on the relative contents of volatile components. A comparative analysis revealed that teas treated by the different drying methods contained the same constituents; these compounds were further classified into six categories and are presented in Table 4. A total of 93 volatile components were detected, only 31 volatile components showed significant differences including twelve kinds of aldehydes, five kinds of alcohols, two kinds of ketones, three kinds of alkenes, four kinds of esters, and five kinds of other substances, among which alcohols and aldehydes were the main compounds.

Upon further analysis, compared with the HR drying, the HA drying increased contents of phenyl acetaldehyde, decylaldehyde, linalool, *trans*-oct-2-en-1-ol, heptan-1-ol, and methyl salicylate. The above substances had a positive correlation with the sweet and flowery flavor of black tea [9,14,24], while the HR drying increased the contents of 3-methylbutyraldehyde, 2-methylbutyraldehyde, hexanal, hexyl hexanoate, *cis*-3-hexene, *trans*-geranyl acetone, and *β*-ionone, which had a positive correlation with the fruity flavor of black tea.

### 3.4. Comparison of the Metabolic Profiles of the HA and HR Drying Methods

**Figure 2:**
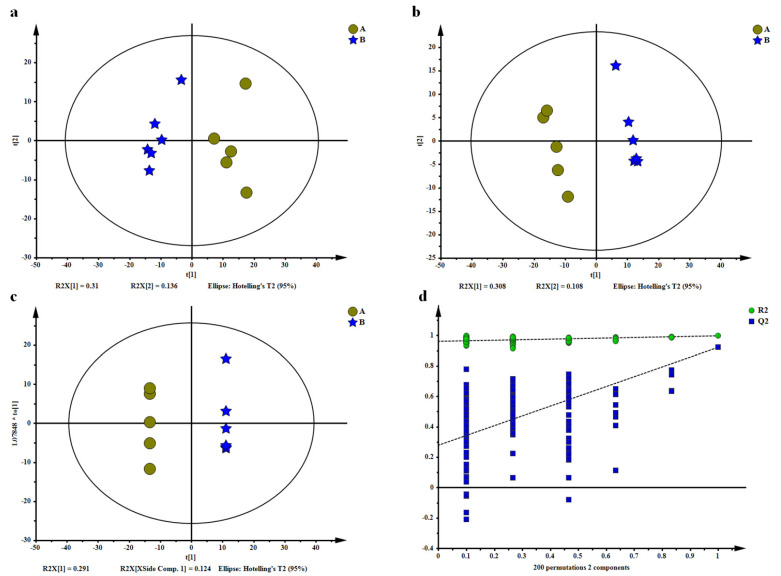
(**a**) Score plots from principal component analysis. (**b**) Partial least squares discrimination analysis. (**c**) Orthogonal partial least squares discrimination analysis. (**d**) Its permutations test. The dataset comprised 966 filtered ion features. Stars in blue show samples from the HR treatment group, spots in yellow indicate the samples from the HA treatment group, Stars in blue show samples from the HR treatment group, spots in yellow indicate samples from the HA treatment group.

The PCA scores plot (R^2^X = 0.446, Q^2^ = 0.0902), PLS-DA scores plot (R^2^X = 0.416, R^2^Y = 0.997, Q^2^ = 0.922), OPLS-DA scores plot (R^2^X = 0.674, R^2^Y = 1, Q^2^ = 0.945) and permutation test showed high reproducibility, reliability of the metabolomics results, and clear distinction between the two drying methods (Figure 2). The result also showed an evolving pattern, which indicated that different drying treatments influenced the chemical profile of Yihong Congou black tea. A total of 966 features were collected in this experiment, and a total of 17 differential metabolites with significant differences were identified in HA drying samples compared to HR drying samples, among these metabolites, five upregulated and 12 downregulated, based on the criteria of VIP > 1, *p* < 0.05.

**Figure 3:**
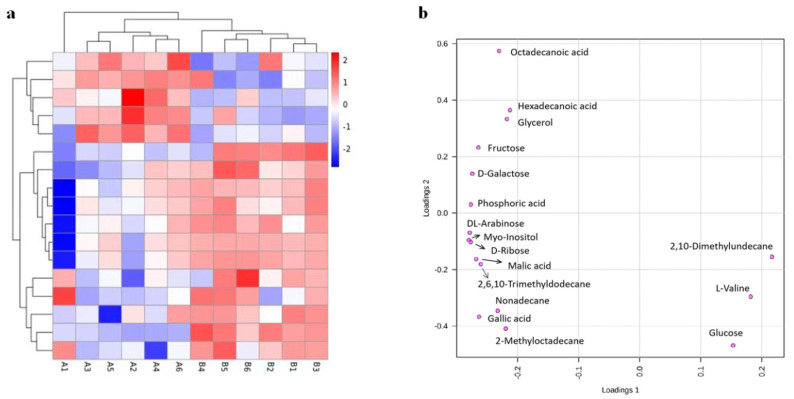
(**a**) Heat map of metabolites in tea samples dried by the HA and HR treatments. Group A, Yihong Congou black tea processed by HA drying; Group B, Yihong Congou black tea processed by HR drying. Warm color and cold color indicate increased and decreased expression of the metabolites, respectively. (**b**) Loading plot of metabolites in tea samples dried by the HA and HR treatments, based on the criteria of VIP > 1, *p* < 0.05, and match score ≥ 700. D-ribose and gallic acid were considered as the most influential characteristic metabolites.

The differential metabolites are shown in Table 5, where five free amino acids showed extremely significant changes in the two drying methods. The levels of most free amino acids including hexadecanoic acid, phosphoric acid, malic acid, octadecanoic acid, and L-valine significantly increased in the HA drying method compared to the HR drying method, while the levels of phenylalanine, proline, and glutamic acid stayed relatively stable. Regarding soluble sugar, D-galactose, fructose, and glucose decreased, but D-ribose and DL-arabinose increased in the HA drying treatment in comparison with the HR drying treatment. According to the Kyoto Encyclopedia of Genes and Genomes (KEGG) analysis, the main metabolic pathway of the 17 differential metabolites were involved in sugar metabolism, fatty acid metabolism, and amino acid metabolism. As is known, the above three disturbed metabolic pathways were related to the taste and aroma. Hierarchical clustering of the differentiated metabolites are shown in the heat map (Figure 3a).

To better overview the changes in the differential metabolites of Yihong Congou black tea treated through the two different drying methods, lording plot (Figure 3b) analysis of different drying treatment reflected that D-ribose, gallic acid, D-larabinose, 2-, 6-, 10-trimethyldodecane, and myo-inositol were the main components corresponding to the hot-air drying black tea sample, while D-galactose, fructose, glucose, malic acid, hexadecanoic acid, L-valine, 2-methyl-octadecane, octadecanoic acid, glycerol, and phosphoric acid were the main components corresponding to the hot-roller drying black tea sample. Therefore, D-ribose and gallic acid were the main characteristic metabolites of the hot-air dried Yihong Congou black tea.

## 4. Discussion

In this study, Yihong Congou black tea was produced from *C. sinensis* (L.) O. Kuntze cv. E’cha NO1, which has strong bud-breeding ability, vigorous growth, abundant contents, and obvious yield advantages. Thereafter, the effect of HR drying and HA drying on the sensory evaluation, chemical quality, antioxidant activity, and metabolic profile of Yihong Congou black tea was investigated.

### 4.1. Drying Methods Affects Sensory Scores, Chemical Qualities, and Antioxidant Capacities of Black Tea

Sensory evaluation revealed that HA drying could better retain the color of dried Yihong Congou black tea than HR drying. This phenomenon had also been observed in other materials using these two drying methods [25,26]. For example, green tea dried by HR was darker than those dried by HA [27,28]. The underlying mechanism of this difference in color darkening of Yihong Congou black tea could be due to the heating effect during drying, causing non-enzymatic greying reactions that depend on the heating temperature and time. Therefore, the HA dried tea obtained a brighter color than the HR dried tea as the temperature of the HA drying was lower than that of HR drying.

As is known to all, tea has many functions such as anti-oxidation, hypolipidemic, and anticarcinogenic activities due to its polyphenols and amino acids, which are the important chemical components in tea and the main components of the fresh and brisk taste of liquid [25,29,30]. In this study, the HA treated tea showed a significantly higher value in water extracts, amino acids, polyphenol, theaflavins, thearubigins, theabrown content, and DPPH free radical scavenging rate than that of the HR treated tea. Researchers Duvivier and Prathapan both found that higher drying temperature caused greater losses of total phenolic contents [24,25]. Furthermore, tea polyphenols are predominant catechins, which can be oxidized to form theaflavin, thearubigin, and theabrown under enzyme catalysis in the fermentation process of black tea [12]. As above-mentioned, the temperature of the HR drying was higher than that of the HA drying, therefore, our finding is consistent with the previous study.

### 4.2. Drying Methods Affects Aroma of Black Tea

Tea volatiles are important components of tea aroma, which has a great impact on the sensory quality of tea. The HA dried tea increased the volatile contents that had positive correlation with the sweet and flowery flavor of black tea, while the HR dried tea increased contents that had a positive correlation with the fruity flavor, probably due to the unique heating conduction by HR drying. It was likely that HA drying could increase the contents of glucoside alcohols, resulting in a decrease in alcohol content and might be a good accelerator of the formation of sweet flavors. Under the action of high temperature heating, these components in HR treated tea undergo hydrolytic reactions and turn into a free state and give out a flowery, fruity note [27]. Thus, the drying process contributed to the formation of the Yihong Congou black tea aroma.

### 4.3. Drying Methods Affects Metabolic Profile of Black Tea

Non-targeted metabolomics indicated that the levels of most free amino acids significantly increased, while the levels of most soluble sugars were reduced in the HA drying method compared to the HR drying method. These data were consistent with the tea physico-chemical quality and analysis of the volatiles. Generally considered, amino acids contribute mostly to the sweet, mellow, and umami flavor of tea infusions [31]. These could contribute to the sweet aroma of Yihong Congou black tea treated through HA drying. In addition, Yihong Congou black tea treated by HR drying had a fruity flavor and higher soluble sugar, which may be because of the Maillard reaction during high temperature in the HR drying process. These could explain the aroma of the final made black tea.

## 5. Conclusions

In the present study, the effects of two different drying methods on the sensory evaluation, chemical quality, antioxidant activity, and metabolic profile of the Yihong Congou black tea process from Ethe cha 1 variety were investigated. The Yihong Congou black tea dried with HA obtained higher sensory scores, better chemical qualities, and higher antioxidant capacities compared to that dried with HR. Moreover, the HA drying black tea had a sweet and flowery flavor, while the HR dried black tea had a fruity flavor. The results of the non-targeted metabolomics showed that the HA dried tea had higher levels of most free amino acids and a lower level of most soluble sugars compared to the HR dried tea, which further confirmed the above experimental data and illustrated the mechanism underlying the formation of black tea taste through the two drying methods. Our study provides a novel characterization of Yihong Congou black tea taste formation and is beneficial to promoting the quality control of Yihong Congou black tea during processing.

## Figures and Tables

**Table 1:** Sensory quality scores of the Congou black tea treated with different drying methods.

Samples	Dry Tea Color (10%)	Dry Tea Streak (10%)	Liquor Color (10%)	Aroma (30%)	Taste (30%)	Infused Leaf (10%)	Total Scores
HA	90 ± 0.5a	86 ± 1.0b	91 ± 0.5a	89 ± 0.6a	89 ± 0.5a	89 ± 0.8	89.0 ± 0.6a
HR	88 ± 0.7b	90 ± 0.8a	89 ± 0.6b	87 ± 0.4b	85 ± 0.7b	88 ± 0.5	87.1 ± 0.4b

Note: HA, hot air dried Yihong Congou black tea; HR, hot roller dried Yihong Congou black tea. Data are presented as mean ± standard deviation (*n* = 5). Mean values with the different lower case letters in the same column indicate significant difference with the least significant difference (LSD) test (*p* < 0.05).

**Table 2:** Results of the color qualities of the Congou black tea treated with different drying methods and the results of the color qualities of broken green tea by different processes.

Samples	Tea Color		Brew Color		Infused Color	
	*L*	*b*/*a*	*L*	*b*/*a*	*L*	*b*/*a*
HA	15.65 ± 0.09 A	1.97 ± 0.02 B	72.50 ± 0.08	4.03 ± 0.02 B	18.19 ± 0.50 A	1.63 ± 0.01 B
HR	15.00 ± 0.13 B	2.13 ± 0.09 A	72.43 ± 0.09	4.10 ± 0.01 A	16.37 ± 0.39 B	1.73 ± 0.02 A

Note: HA, hot air dried Yihong Congou black tea; HR, hot roller dried Yihong Congou black tea. *L* represents lightness, the higher the value, the brighter the color, and *b*/*a* represents hue angle, the lower the ratio, the redder the color. Data are presented as mean ± standard deviation (*n* = 5). Mean values with the different capital letters in the same column indicate significant difference with least significant difference (LSD) test (*p* < 0.01).

**Table 3:** Physico-chemical quality of the Congou black tea treated with different drying methods/%.

Samples	Water Extracts	Amino Acids	Polyphenols	Soluble Sugar	TF_S_	TR_S_	TB_S_
HA	29.10 ± 0.44 a	3.01 ± 0.04 A	13.28 ± 0.20 A	6.06 ± 0.20 b	0.40 ± 0.03 a	4.61 ± 0.15 a	9.35 ± 0.40 A
HR	27.46 ± 0.23 b	2.84 ± 0.00 B	9.09 ± 0.11 B	6.69 ± 0.18 a	0.35 ± 0.01 b	4.06 ± 0.12 b	7.67 ± 0.15 B

Note: HA, hot air dried Yihong Congou black tea; HR, hot roller dried Yihong Congou black tea. Data are presented as mean ± standard deviation (*n* = 5). Capital letters (A, B) and lower case letters (a, b) represent a statistically significant difference within each column at *p* < 0.05 and *p* < 0.01, respectively.

**Table 4:** Main volatile compounds of the Congou black tea treated with different drying methods.

Category	Odorant	Odor Perception	Content (µg/L)	
			HA	HR
Aldehydes	3-Methylbutyraldehyde	Fruity, Sweet	0.071	0.32
	2-Methylbutyraldehyde	Fruity	0.20	0.54
	Hexanal	Grassy green, Fruity	1.82	3.08
	2-Hexenal	Grassy green	7.11	8.86
	Heptanal	Grassy green	0.66	1.04
	Benzaldehyde	Bitter almond flavor	4.44	3.99
	Phenyl acetaldehyde	Floral	12.52	6.54
	*Trans,trans*-2,4-heptadienal	Grassy green	1.96	0.84
	*Trans*-oct-2-enal	Fatty taste	0.63	1.27
	*Trans*-2-nonenal	Rose	0.13	0.25
	Decanal	Sweet	0.80	0.52
	β-Cyclocitral	Lemon flavor	2.35	2.57
Alcohols	Linalool	Bell orchid fragrance	19.10	14.88
	Himachalol	Woody	2.19	2.52
	*Trans*-terpinen-4-OL	Floral	0.051	0.12
	*Trans*-oct-2-en-1-ol	Floral	0.66	0.15
	Heptan-1-ol	Fatty taste, spicy	0.26	0.12
Ketones	*Trans*-geranyl Acetone	Fruity, Sweet	0.78	1.12
	β-ionone	Woody, Fruity	2.28	3.53
Alkenes	Limonene	Sweet	0.09	0.41
	β-pinene	Woody	0.41	0.17
	4,7,7-Trimethylbicyclo hept-2-ene	Camphor odor	0.97	1.02
Esters	Methyl hexanoate	Ether odor	0.021	0.025
	Hexyl hexanoate	Fruity	0.35	0.41
	Methyl salicylate	Sweet	22.01	15.66
	*Cis*-3-hexene	Grassy green, sweet, fruity	1.29	1.33
Other compounds	Dimethyi sulfide	Aroma of new tea	0.05	0.13
	2-Methyl furan	Ether odor	0.20	0.04
	Toluene	Benzene odor	0.031	0.082
	Naphthalene	Floral	0.16	0.30
	α-Methylnaphthalene	Floral	0.19	0.19

**Table 5:** The significantly different metabolites between the different drying methods.

No.	RT/min	Name	Match	VIP	*P*/*T*-Test	log2FC_HA/HR
1	18.354	Hexadecanoic acid	926	1.1281	0.0470	−0.2581
2	16.420	D-Galactose	925	1.7667	0.0000	−0.1467
3	16.127	Fructose	922	1.4236	0.0058	−0.1508
4	16.441	Glucose	920	1.4569	0.0041	−0.6999
5	8.803	Phosphoric acid	901	1.2672	0.0204	−0.1303
6	11.669	Malic acid	901	1.3235	0.0136	−0.1000
7	18.862	Myo-Inositol	900	1.5149	0.0021	0.0850
8	8.798	Glycerol	896	1.1849	0.0343	−0.2474
9	17.154	Gallic acid	895	1.7217	0.0000	0.2589
10	21.070	Octadecanoic acid	895	1.2083	0.0298	−0.2691
11	7.938	L-Valine	871	1.6172	0.0005	−1.0702
12	5.579	2,6,10-Trimethyldodecane	863	1.3412	0.0119	0.6830
13	19.361	Octadecane, 2-methyl-	855	1.2689	0.0202	−0.1432
14	14.244	Nonadecane	835	1.1689	0.0376	−0.1294
15	22.321	2,10-Dimethylundecane	774	1.4382	0.0050	−0.7777
16	28.313	D-Ribose	754	1.2642	0.0209	0.0555
17	27.430	DL-Arabinose	700	1.2362	0.0251	0.0764

Note: FC is fold changes described with the log2 transformed numbers. Positive values of FC indicate upregulation, and negative values indicate downregulation.

## Data Availability

Not available.

## Sample Availability

Samples of the Yihong Congou black tea dryed by hot roller and hot air are available from the authors.
